# Co-creation of a motor–cognitive exercise programme—a qualitative study with older people and physiotherapists

**DOI:** 10.1186/s12877-025-06522-3

**Published:** 2025-10-15

**Authors:** J Hallin, A Arola, M E Domellöf, M Zingmark, M E Taylor, M Sandlund, A Toots

**Affiliations:** 1 Department of Community medicine and rehabilitation, Umeå University, Umeå, Sweden; 2 Arcada UAS, Helsinki, Finland; 3 Department of Psychology, Umeå University, Umeå, Sweden; 4 Östersund, Municipality of Östersund, Sweden; 5 School of Health Sciences, UNSW Sydney, Sydney, NSW Australia; 6 Falls, Balance and Injury Research Centre, Neuroscience Research Australia, Sydney, NSW Australia

**Keywords:** Aged, Balance exercise, Dual task, Falls prevention, Participatory research

## Abstract

**Background:**

To reduce the risk of falls, tailored interventions including exercise that simultaneously challenges cognition (motor–cognitive) are recommended. However, considerable variation in motor–cognitive approaches exist, and its use in clinical practice is less widespread. This study aimed to explore older peoples’ and physiotherapists’ perspectives on motor–cognitive exercise and their suggestions for programme development during co-creation.

**Method:**

Community-dwelling women (*n* = 8) and men (*n* = 9), aged (mean ± SD) 74 ± 5.6 years, and 4 physiotherapists working in geriatric rehabilitation were included. Data were collected through nine workshops. The discussions were audio-recorded and analysed employing a qualitative content analysis approach. This study aligns with the Consolidated Criteria for Reporting Qualitative Research (COREQ) checklist.

**Results:**

The analysis resulted in four themes and nine categories. The themes were: *discovering the motor–cognitive concept through engagement in activity*, *balancing safety and challenge*, *navigating the complexity of individualisation*, and *managing motivation and compliance*. The context was initially difficult to grasp. Performing practical activities led to understanding, and motor–cognitive exercises were experienced as enjoyable yet challenging to perform. Balancing safety while providing exercises that were challenging enough to make the programme effective was perceived as challenging by both older participants and physiotherapists. Regular individual follow-up during the exercise programme was considered important for promoting progression and compliance. Leader-led, group-based exercises later incorporated into daily life routines were suggested to support motivation and promote safety.

**Conclusion:**

Incorporating motor–cognitive exercise in fall prevention exercises programmes for older people at risk of falls, may enhance enjoyment and motivation but requires consideration for safe and effective delivery. The co-creative design in this context is rare and the results may be useful for further research and for the development of fall prevention interventions.

**Clinical trial number:**

Not applicable.

**Supplementary Information:**

The online version contains supplementary material available at 10.1186/s12877-025-06522-3.

## Introduction

Falls are a frequent cause of injury in older people [1], leading to short- or long- term disability, reduced independence, and an elevated risk of mortality [2]. Older people who have experienced falls often report pain, injury, social and daily activity limitations, and feelings of fear and helplessness [3]. The aetiology of falls is often multifactorial, encompassing many non-modifiable and modifiable causes and risk factors. While falls can occur at any age, risk escalates with advancing age [1]. Ageing is associated with increased risk of physical and cognitive impairments and chronic medical conditions [4, 5], with reduced muscle strength, balance, and cognition being risk factors for falls [6–8]. Injuries related to falls impose substantial burdens on individuals, healthcare systems, and societal economies [1]. Consequently, falls among older people represent a major public health concern, underscoring the need for further research into effective and sustainable fall prevention interventions [1].

Physical exercise has been shown to be an effective fall preventive intervention for older people [9–13], and interventions incorporating strength, balance, and functional exercises have been proven to reduce the rate of falls by 24%–34% and decrease the risk of falls by 13%–22% [14]. For older people at intermediate risk of falls motor–cognitive—also called ‘dual-task’—exercises are recommended in addition to physical exercise [9]. Dual-task exercise combines physical exercise with cognitive tasks, and during dual-task exercises, physical and cognitive tasks are performed simultaneously [15]. Incorporating a combination of physical (motor) and cognitive components into exercise programmes for older people has shown promising effects on important fall risk proxies, such as cognitive function, walking, and balance [15, 16], as well as improved performance in multicomponent daily tasks [15]. However, there is considerable variation among motor–cognitive approaches; for example, the selection of motor–cognitive tasks, as well as their duration and frequency [15]. Furthermore, many of the established falls prevention programmes used today do not incorporate motor–cognitive exercises [17].

Developing health interventions through co-creation is recommended to ensure relevance for the target population, increase confidence in the results, and expedite translation into practice; leading to more immediate and significant outcomes and impact [18, 19]. Therefore, we developed a motor–cognitive exercise intervention for older people at risk of falls using co-creation. When developing fall prevention exercise programmes, a range of factors must be considered; e.g., the older person’s fall history, context, resources, and the personal goals and values related to activities that the person considers meaningful [9]. Motivations for engaging in exercise vary; for some, the act of exercising itself is sufficient, while others require tangible benefits, such as being able to participate in meaningful activities [20]. Hence, it is important to acknowledge the diverse values and unique needs of individuals when developing an exercise programme that is feasible and accessible to older people. Additionally, physiotherapists may have differing needs, expectations, and perceptions when prescribing person- centred interventions (e.g., ensuring sufficient challenge for effectiveness while maintaining safety) that are also important to consider. Therefore, the aim of this study was to explore older peoples´ and physiotherapists’ perspectives on motor–cognitive exercise and their suggestions for programme development during co-creation.

## Methods

This study employed a co-creative approach inspired by Participatory and Appreciative Action and Reflection (PAAR) [21]. The PAAR approach is particularly beneficial for knowledge production with the target group and highlights the positive aspects by advocating for a creative and appreciative mindset, instead of focusing on problems [21]. Collected data were analysed using a qualitative content analysis approach [22]. The Consolidated Criteria for Reporting Qualitative Research (COREQ) were utilised (see Additional file 1) when reporting the results to ensure comprehensive reporting and transparency [23].

### Participants

The study included older participants aged ≥ 65 years, residing in ordinary housing within the municipality of Umeå, Sweden. Inclusion criteria were that older participants should be able to independently rise from a seated position without personal assistance, travel to the university campus for workshop participation independently or with assistance, and communicate effectively in group situations. The opportunity to participate with a relative or caregiver was offered to people with mild cognitive impairment or mild dementia. Exclusion criteria were: having a diagnosis of moderate to severe dementia or being in palliative care.

### Recruitment

Older participants were recruited through senior services and senior citizens in Umeå, with information distributed through presentations and advertisements. Individuals with access to relevant email lists and networks were contacted to disseminate information about the study to potential participants. Only participants who were interested in participating contacted researchers voluntarily to provide their contact details. We sought 24 older participants to fill two concurrent workshop groups of approximately 10–12 older participants each, allowing for potential attrition. An online form with project information and what participants could expect was disseminated, through which interested people in the project could register. Older participants were included based on registration order, while also aiming to maintain age and gender balance. Four physiotherapists who were active practitioners within Region Västerbotten or Umeå municipality with experience in geriatric rehabilitation were recruited through the authors’ professional network.

### Data collection

Prior to the first workshop, a brief telephone interview was conducted to collect background information such as age, sex, living arrangement, previous occupation and history of falls within the previous year. Furthermore, data were collected on self-reported independence in daily activities [24], physical activity level [25] and health, using the first question from the Short-Form-36 Health Survey [26]. Participants also responded to questions concerning memory, concentration, learning and attention, as well as engagement in cognitive stimulating activities. Before the first workshop commenced, two older participants announced that they could not participate in the project due to health-related concerns. During the study, an additional five older participants elected to end their participation, citing reasons such as personal or familial health-related concerns (*n* = 3), or unknown reasons (*n* = 2). Subsequently, the older participant group consisted of community-dwelling women (*n* = 8) and men (*n* = 9), with an average age of 73.7 years (Table 1). Participant characteristics are reported in Table 1. Most older participants were independent in their instrumental activities of daily living (IADL) [24]. Two-thirds of the older participants had experienced one or more falls in the previous year. None of the participants reported experiencing difficulties related to memory, concentration, learning or attention. Moreover, all actively engaged in cognitively stimulating activities, e.g. crosswords, handicraft or computer games. Additionally, four practicing physiotherapists within Region Västerbotten (*n* = 2) and Umeå municipality (*n* = 2), with a range of 1–29 years of experience in geriatric rehabilitation, were recruited and participated in the workshops. Data (notes, audio recordings) were collected during discussions and other interactive activities in the workshops.

**Table 1:** Characteristics of participants

Characteristics	Older participants (*n* = 17)	Physiotherapists (*n* = 4)
Age, years, mean ± SD (range)	73.7 ± 5.6 (66–83)	42.5 ± 13 (24–53)
Women, n (%)	8 (47.1)	4 (100)
Independent in Instrumental Activities of Daily Living n (%)	16 (94.1)	
Lives alone, n (%)	7 (41.2)	
Tertiary educational level n (%)^2^	9 (53)	
Secondary educational level n (%)^1^	8 (47)	
Self-rated health; good, very good or excellent, n (%)	15 (88.2)	
Fallen previous year, n (%)	11 (64.7)	
Physical activity, moderate intensity *≥* 30 min/week, n (%)	8 (47.1)	
Physical activity, low intensity *≥* 30 min/week, n (%)	16 (94.1)	
Experience in geriatric rehabilitation, years, mean (range)		15 (1–29)

^1^ Based on International Standard Classification of Education 2011 [27]

SD = standard deviation

### The workshops

Workshops are a creative and hands-on participatory approach to generating and gathering reliable and valid data [28], and the workshops in this study were structured and planned by authors AA, MD, MET, MS and AT, based on an iterative design encompassing planning, action, reflection, and evaluation [29]. Additional co-creation principles were incorporated to enhance group creativity and productivity, such as ownership and control (Table 2) [29]. During the workshops, moderators and the participants aimed to foster an environment conducive to open discussion, and the moderators facilitated discussion by asking questions and offering input and reflections. A summary of the group discussions and conclusions were presented towards the end of every workshop, as a form of member-checking to increase face validity.

**Table 2:** An overview of the workshop structure and description of the content of each workshop

Workshop (month)	Content	Participants
1 (Feb)	**Aim**: Introduce the project, workshop theme, and co-creation principles. **Up- skill**^**1**^: Falls and falls prevention, balance exercise, cognition, and cognitive exercise. **Activity**: Group discussions, experience sharing. **Home assignment**: Reflect on use of motor–cognitive abilities in daily life activities.	Group 1a: Older participants *n* = 10 Group 1b: Older participants *n* = 6
2 (Feb)	**Aim**: Explore experiences and preferences of motor–cognitive exercise. **Up- skill**: Motor–cognitive exercise. **Activity**: Group discussions, test early prototypes of motor–cognitive exercises. **Home assignment**: Reflect, together with friends or family, on motor–cognitive exercise, including motivating factors, feasible in daily life.	Group 2a: Older participants *n* = 5 Group 2b: Older participants *n* = 5
3 (Mar)	**Aim**: Introduce the project, workshop theme and co-creation. **Up- skill**: Falls, falls prevention, and motor–cognitive exercise. **Activity**: Group discussion, sharing experiences.	Physiotherapists *n* = 3
4 (Mar)	**Aim**: Explore mode of delivery and packaging **Activity**: Testing different modes (video, audio, text, in real life), group discussions, voting for preferred mode.	Group 4a: Older participants *n* = 6 Group 4b: Older participants *n* = 3
5 (Apr)	**Aim**: Apply motor–cognitive exercises to a wider context within falls prevention, including older people with various function. **Activity**: Group discussions around clinical cases.	Older participants *n* = 13 and Physiotherapists *n* = 2
6 (Dec)	**Aim**: Refine instructions and performance of motor–cognitive exercises. **Activities**: Test prototypes of motor–cognitive exercises, group discussions.	Older participants *n* = 9

^1^ Up-skilling is a strategy in the co-creating process intended to give the participants necessary knowledge to be able to contribute to the co-creation in an active way

Co-creation involved six workshops on Umeå University campus (Table 2). The older participants were invited to attend five workshops in total. For workshops one, two, and four they were divided into two groups, with workshops scheduled at different times. Physiotherapists attended the third workshop alone and joined the older participants in the fifth workshop. Overall, 17 older people and 4 physiotherapists participated across the six workshops. Three to five researchers from the project group participated in the workshops, assuming the role of moderators (JH, AA, MD, MS, and AT). Each workshop lasted approximately 2.5 h, and light refreshments were served during the workshops. The workshops were spaced a few weeks apart, except for the interval before the last workshop, which was six months. During these six months the moderators refined and developed a prototype of the motor-cognitive exercise programme, which was subsequently tested in a group setting during the sixth workshop by the older participants. Moreover, the time between workshops allowed the older participants, physiotherapists, and moderators to reflect, develop ideas, and prepare materials; e.g., after workshops one and two, the older participants received take-home assignments to reflect upon (Table 2).

### Workshop activities

Between workshops, moderators analysed participants’ evaluations, notes, and recordings to adjust the content for the upcoming sessions, ensuring workshops were linked to previous discussions and facilitating the co-creative process. For example, activities which included early prototypes of exercises were added in the second workshop because the moderators discerned that participants expressed difficulties grasping the motor–cognitive concept. AT and MD developed prototype exercises and planned for the next workshop according to discussion evaluations from previous workshops. The motor-cognitive prototypes included balance exercise such as stepping, turning, or walking with a narrow base of support, performed simultaneously with cognitive tasks. These cognitive tasks involved verbal fluency (e.g., naming items within a category) and serial subtraction (e.g., counting backwards by twos or threes). Executive function tasks targeted inhibition, shifting, and updating [30]. Inhibition involved withholding movement in response to specific cues; shifting required alternating between different instruction sets; and updating entailed recalling and executing the final movement in a sequence. The six months interval between workshops five and six was intentionally scheduled to allow time for researchers to reflect and discuss results from the workshops and to develop and refine the prototype exercises (AA, MD, MET, MS, JH and AT).

### Data management and analysis

Audio recordings from workshops were analysed using qualitative content analysis to allow for concentrated focus on the content and contextual meaning of the communication [22] using MAXQDA [31] software. After the last workshop, one author (JH) repeatedly listened to the recordings and transcribed them verbatim to become familiar with the data, before dividing the interviews into meaning units and coding them independently. The codes were compared for similarities and differences and clustered into preliminary subcategories that were further clustered into nine categories. The categories were then abstracted and merged, resulting in four themes that described the latent content. The analytic process from codes, subcategories, categories and themes were initiated by JH, then iteratively discussed in smaller author groups (AT, MS) before being finalized in discussions with the whole research group. Furthermore, the authors went back and forth between the codes, categories, and themes and back to the transcribed texts. In this way, the data was triangulated by drawing on preunderstanding derived from various prior experiences and professional backgrounds in the author group. An example of the analytic process is presented in Table 3, while a more detailed overview tracing the progression from codes to themes is available in Additional file 2.

**Table 3:** Example of the analytic process

Meanings unit	Code	Subcategory	Category	Theme
PW^1^: Well, I´ve thought about it until my brain hurts, but I can´t come up with anything else. When it comes counting and stuff like that, like running and calculating at the same time, it´s not something you´d think of on your own. But I don´t know…	Difficult to come with examples of motor–cognitive exercises.	Motor–cognitive exercises are difficult to describe.	To grasp a new concept can be challenging	Discovering the motor–cognitive concept within activities
PM^2^: I hadn´t really thought about that before, but it was when I saw this project that I started considering it. Like, maybe it´s helpful for the memory if you add some movement, or vice versa.	Not previously reflected on motor–cognitive activities in everyday life	Awareness of motor–cognitive exercise is low		
PM: I found it really useful, and quite fun too. Then we also counted backwards…I thought it was stimulating.	Counting backwards was stimulating	Motor–cognitive activities are meaningful	Practice leads to enlightenment	
PW: Yes, that was the hardest part. PW: Yes, when you skipped one and when I made a mistake once, it was like a short circuit. Everything went wrong. Now I´ve made a mistake, I have to start over, but you kept going…	One mistake in an inhibition exercise and everything went wrong	Motor–cognitive exercises was difficult		

^1^PW = Older participant Woman

^2^PM = Older participant Male

### Authors background

All authors except MD and MS have experience working within geriatric care in different professions and contexts. MD is an experienced researcher focusing on cognition and cognitive support for older people from different clinical populations in Sweden. AT, MS, and MET are physiotherapists with clinical experiences of fall preventive interventions for older people in different contexts: AT in England and Sweden, MS in Sweden and Norway, and MET in Australia. JH, AA, and MZ are occupational therapists with clinical experiences of fall preventive interventions for older people in different contexts: JH and MZ in Sweden, and AA in Finland and Sweden. Furthermore, several of the authors (AT, MS, MET, MD, MZ and AA) have experience in qualitative research, co-creation, and research concerning balance exercise, falls and fall prevention. MD, MET, and AT have experience in evaluating cognitive training and/or dance in clinical groups, and the connection between cognition and motor skills in older people and clinical groups. In addition, all authors have pedagogical expertise.

### Trustworthiness

Trustworthiness is described in terms of credibility, dependability, and transferability in studies with a qualitative approach [22]. We aimed to recruit participants with diverse experiences in fall prevention exercises for older people, including both older people and physiotherapists. The older participants recruited were relatively young and physically active, which may be a limitation. Furthermore, the authors come from different disciplines and brought different perspectives to the study, which enriched the analysis and contributed to its credibility. Dependability was ensured by discussions and reflections during the workshops, between the workshops, and after the workshops ended. These reflections guided the researchers in the subsequent data analysis. Moreover, during the data analysis, the authors reflected regularly, both together and separately, on possible interpretations. To enable transferability of the results, both the characteristics of the older participants and the physiotherapists were described. Additionally, quotations from the older participants and the physiotherapists add richness to the description of the participants’ experience of the co-creation of the motor–cognitive exercise programme. The context of this study, conducted in a city in northern Sweden, may limit the transferability of the findings both nationally and internationally.

### Ethical consideration

The study follows the ethical principles outlined in the Declaration of Helsinki. Ethical approval was granted from the Swedish ethical review authority (Dnr 2022–05826-01.). Participants received detailed written information outlining the study and the scope of participation, including privacy protection in data handling and the right to withdraw at any point without explanation or consequences, and provided written consent to participate.

## Results

The analysis of the older participants’ and physiotherapists’ perspectives and reflections on motor–cognitive exercises during the co-creation process resulted in four themes: *discovering the motor–cognitive concept through engagement in activity*,* balancing safety and challenge*,* navigating the complexity of individualisation*, and *managing motivation and compliance* (Table 4) and below each theme is described with its respective categories and is illustrated by quotes from the workshops’ audio recordings.

**Table 4:** An overview of categories and themes

Category	Theme
To grasp a new concept can be challenging	Discovering the motor–cognitive concept through engagement in activity
Practice leads to enlightenment	
To effectively challenge balance is inherently unsafe	Balancing safety and challenge
Safety is a crucial aspect	
It is important to recognise variability within the older population	Navigating the complexity of individualisation
Multiple components complicate progression	
To exercise together for enjoyment and social engagement	Managing motivation and compliance
To maintain motivation through meaningful exercise and structure	
To establish routines requires extra support	

### Discovering the motor–cognitive concept through engagement in activity

The categories within this theme pertain to participants’ journey of assimilating knowledge and comprehension of this novel concept, a prerequisite for in-depth discussions. At first, participants struggled to understand and integrate the context, so it was important to give enough time and focus to this basic component. Engaging in practical, hands-on activities and testing the prototypes expedited learning, leading to enhanced recognition of the concept of interest and new perceptions of the participants’ own abilities.

### To Grasp a new concept can be challenging

The motor–cognitive concept was novel to the older participants, and initially, they encountered challenges in comprehending the concept. Therefore, exploring past experiences and providing examples of motor–cognitive activities in everyday life was initially expressed as difficult by the older participants. After discussing the concept together, the older participants were able to share experiences from their own contexts, which made the concept more concrete. For example, they described various sports that they currently engaged in or had previously practiced, such as alpine skiing, yoga, or ball sports as motor–cognitive activities. Additionally, several older participants described cognitive strategies employed in everyday life activities; for example, memorising grocery shopping lists based on the layout of the grocery store. They concluded that shopping in this manner could be considered a motor–cognitive activity. Through collaborative discussions and sharing experiences, they were able to identify more experiences. For instance, they recognised that walking in a busy area while using headphones, or talking with a friend, or even just navigating uneven terrain in the forest, walking the dog, or hunting might constitute a motor–cognitive challenge. Despite initially struggling to pinpoint concrete examples, the older participants acknowledged that they likely engage in numerous motor–cognitive activities in their daily lives without consciously realising it.

*“I hadn’t really thought about it*,* it was just when I saw this project that I started pondering it*,* specifically the combination of…that yes*,* perhaps it enhances memory when one has a bit of movement*,* or vice versa.”* (male older participant, workshop 1).

Dancing with a partner was another activity regarded as a motor–cognitive activity. One group reasoned that if you can dance without conscious thought, your dual-task ability is good. Conversely, in their experience, less skilled dancers often pause their dancing when conversing with their dance partner. The older participants also expressed awareness of the risks associated with engaging in activities that pose a high motor–cognitive challenge. For example, stepping down a sidewalk edge while simultaneously looking out for traffic was perceived as a demanding task. The physiotherapists knew of the concept but had limited or no prior experience of using motor–cognitive exercise in their practice. Some physiotherapists had themselves utilised motor–cognitive exercises, or were aware of colleagues who had done so, such as walking while simultaneously tossing a ball in the air.

### Practice leads to enlightenment

When the older participants engaged with prototypes of motor–cognitive exercises, the concept became clearer to them. The experience of performing the prototyped motor–cognitive exercises was both enjoyable and meaningful for the older participants. Although they found the exercises beneficial, some were challenging to perform and led to new perceptions of their own abilities.

*“I found it interesting to discover that I couldn’t do things I thought I would be able to.”* (male older participant, workshop 2).

Some concerns were expressed by the older participants in relation to performing the motor–cognitive exercises; for example, following exercise instructions through headphones while performing them alone in a public setting could be embarrassing. Furthermore, the older participants experienced that they prioritised the balance task over the cognitive task when faced with more challenging activities. The physiotherapists also enjoyed the motor–cognitive exercises but were concerned they might be too advanced for patients with severely impaired balance. Therefore, they emphasised the importance of targeting the appropriate population for this type of exercise. Also, the physiotherapists believed these exercises could be practical, useful, and easily integrated into patients’ daily activities. Developing and utilising motor–cognitive exercises in practice was described as being of the utmost importance.

*“Being able to stand and let go and do something while being disturbed*,* like if someone calls out or the phone rings*,* it is very transferable*,* regardless of function or starting point in terms of capacity.”* (physiotherapist, workshop 3).

### Balancing safety and challenge

The categories within this theme encompass the challenge of engaging in effective balance exercise unsupervised at home. Safety was paramount for both the older participants and the physiotherapists, albeit from slightly different perspectives.

### To effectively challenge balance is inherently unsafe

Participants discussed the challenge of performing balance exercises as they generally involve a degree of risk. The older participants believed some element of instability was needed for balance exercises to be effective.

*“Of course*,* it should be wobbly to get any effect.”* (female older participant, workshop 4).

The older participants experienced challenging balance exercise as enjoyable, while recognising that many older people have a fear of falling. The physiotherapists shared a similar perspective, emphasising that the exercises must be sufficiently challenging and even somewhat unsafe to qualify as a balance exercise. Furthermore, they stressed the importance of finding exercises that genuinely challenge balance.

### Safety is a crucial aspect

Safety was a frequent topic of discussion among both the older participants and the physiotherapists, albeit from different perspectives. The older participants found what feels safe to be highly individual. They also noted that the importance of security and safety varies among individuals. For example, some disregard safety instructions for exercise machines at the gym, while others believe that clear instructions on how to perform exercises are vital:

*If you’re going to exercise and stand on one leg*,* it’s important to have a stable wall or bench—or something you really trust—preferably on both sides. And you need to know how to start: like making sure you’re properly grounded on the base of your big toe*,* little toe*,* and heel so you’re steady before you begin.* (Female older participant, workshop 4)

The older participants expressed personal responsibility for their safety when exercising at home and discussed strategies; for example, seeking guidance from healthcare professionals to assess safe exercise environments at home or exercising with the assistance of relatives. The importance of safety during physical activity and exercise was emphasised, with older participants providing several examples and these included the use of studded shoes for outdoor exercise during winter to prevent slips on icy roads:

*No*,* because I’ve become really*,* really careful about always wearing studded shoes when it’s snowy. I just don’t go out without them. I mean*,* I can walk to the bus and back*,* but I can’t imagine walking home without my studded shoes.* (Male older participant, workshop 4)

When exercising at home, they highlighted the importance of maintaining a safe physical environment. The use of a rollator was suggested as a potential safety measure to provide support during exercise. However, some older participants had not considered safety when exercising:

*Male older participant 1: I have not thought about that…Hehe…*.

*Male older participant 2: I also haven´t thought about safety. Looking at the gym where I spend quite a lot of time there isn´t anything special there*,* it´s some equipment and stuff that are around you…* (male older participants, workshop 4).

Among the physiotherapists, safety was described as a significant and weighty responsibility. They found that safety often took precedence over effective balance exercises in patients’ homes due to their concern and professional responsibility for their—often frail—patients. The physiotherapists defined their responsibility as based on their professional license, and their understanding and experience of the potential consequences of a fall for their patients. They expressed uncertainty about creating a safe yet challenging motor–cognitive exercise programme for patients to perform independently at home, believing it will be difficult to implement in practice. In their work, they were cautious when prescribing home-based high-challenge balance exercises, preferring safer alternatives such as supported balance exercises. However, they acknowledged that they did not believe such alternatives were ‘real balance exercises’:

*“No*,* you’re very cautious. And is also about ensuring a safe environment for both the patient and the caregiver. So*,* we almost always provide support to prevent any mishaps. Unfortunately*,* that’s how it is.”* (physiotherapist, workshop 3).

Additionally, the physiotherapists reasoned that effective balance exercise likely require some form of supervision to ensure safety. They believed that performing exercises in a facility or clinic, where patients can be supervised, is safer. In addition, a group setting, with a supervisor to observe, instruct, and adjust as needed, could provide an added layer of safety; albeit providing less supervision than one-on-one sessions.

### Navigating the complexity of individualisation

Within this theme, the categories describe the challenge of identifying suitable strategies to promote progression among this highly heterogenous population. This challenge is further compounded by the interplay of the different components within the exercises.

### It is important to recognise variability within the older population

Peoples’ perception of enjoyable physical activities was discussed among the older participants, and they experienced that this perception could change with age. They described this as one reason as to why individual tailored exercise is important. Life-altering events can also influence exercise preferences and needs:

*“I thought that I was the kind of person who wanted to exercise alone*,* but*,* as I said*,* I think I might need the group now to really get started.”* (male older participant, workshop 2).

The older participants emphasised the importance of the layout and design of the programme as one example of variation in preferences. Moreover, they emphasised the need to consider different preferences when designing instructions, progression, and follow-up procedures. Preferences varied, with some favouring supervised sessions, while others found email communication sufficient or needed no follow-up. While some found audio and video instructions helpful during exercise, others considered them overly complex. Interestingly, the consensus among older participants was that paper-based programmes felt out-dated, ineffective, and difficult to read during exercise. They emphasised that such programmes will not be used by older people. However, the physiotherapists experienced variability in using paper-based programmes or digital solutions among their patients, and they emphasised the importance of acknowledging different needs among older people. Because of the variability in the population of older people, progression of the exercise programme was expressed as challenging to manage:

*“Yes…It is really a tough one to solve*,* I mean difficult to organise somehow because people are different…”* (physiotherapist, workshop 3).

Individual preferences can influence task difficulty, with some people, for example, finding tasks like reciting numbers more challenging than reciting letters. Recognising this variability, the physiotherapists proposed beginning with supervised exercise, possibly in a group, and transitioning to unsupervised home-based exercises with follow up.

### Multiple components complicate progression

The concept of progression emerged as important for both older participants and physiotherapists. The exercises involve performing both cognitive and motor tasks simultaneously, which poses challenges for designing and implementing progression strategies addressing these components individually and/or in combination. Therefore, the older participants emphasised the importance of precise instructions to be able to correctly perform the exercises. They expressed the importance of and a commitment to performing the exercises accurately and were aware of the complexity introduced by the dual-task nature of the motor–cognitive exercise. The older participants also expressed their probable need for guidance to ensure correct performance and progression of the exercises:

*“But then*,* when you’ve counted backward to a hundred*,* as you said*,* eventually you didn’t need to think; it just came. So*,* then you must come up with something new. That’s what I was trying to do.”* (female older participant, workshop 5).

To manage these different potential tasks and their progression in a motor–cognitive exercise programme, the older participants highlighted the need for an individually tailored programme as well as individually tailored follow-ups. The older participants expressed that they thought it would be difficult to independently select exercises and manage progression, further highlighting the need for clear information and instructions and support from an instructor to manage progression.

Physiotherapists expressed the fundamental importance of progression, highlighting that without it the potential effects of motor–cognitive exercise will likely be limited. They acknowledged the complexity of managing progression and expressed uncertainty about the best approach, questioning whether to prioritise motor or cognitive tasks and how to prevent memorisation of cognitive tasks. Given the dual-task nature of motor–cognitive exercises, which vary in difficulty, physiotherapists discussed strategies for facilitating progression in clinical practice. They believed patients would require support to manage progression. Despite the tasks’ complexity, physiotherapists agreed that incorporating cognitive tasks into balance exercises is beneficial but requires practical implementation guidance. They stressed that correct execution and clear instructions on progression are essential to prevent falls.

*“I also think about patients with hip fractures who have quite*,* quite good balance but have trouble challenging themselves enough due to pain when weightbearing. But if one can do something with more static exercises with cognitive elements it may be a great tool to add. If you know how to do it*,* which I don´t do today. Other than just by talking or not talking…”* (physiotherapist, workshop 3).

### Managing motivation and compliance

Within this theme, the categories highlight that exercising together, as well as individualised follow-ups, can increase meaning, enjoyment, and motivation. Initially, establishing exercise routines may necessitate additional support.

#### To exercise together for enjoyment and social

The older participants described group exercise as being meaningful and enjoyable in several ways. They appreciated the camaraderie of exercising together, the regularity it brought to their exercise routine, the competitiveness that leads to increased intensity, and the overall enjoyment of the activity. As one older woman described her experienced of group exercise in workshop 2:

*“Yes*,* it makes you feel great. I mean*,* I wasn´t there last week*,* I’ve been sick*,* and now I went on Monday*,* and what a difference it makes! You feel so sprightly when you´ve been*,* it eventually becomes a must to go there.”* (female older participant, workshop 2).

Group exercise led by an instructor was preferred among the older participants. They believed that the instructor could enhance their motivation to exert themselves during the exercise. Furthermore, the older participants felt that group exercise led by an instructor was safer. The social aspect of group exercise was also highlighted as important; not only for fostering social interaction but also for promoting a sense of security. One older male participant discussed this in workshop 5:

*“But this social aspect*,* it´s really the big piece*,* and the content can almost be secondary. That you meet*,* socialise*,* and get some feedback and such things.”* (male older participant, workshop 5).

The older participants shared their experiences of attending group exercises where they cared for each other and engaged in activities outside the exercise, such as enjoying a cup of coffee together. They experienced the social aspect as important and comforting. The physiotherapists echoed the sentiments of the older participants, recounting their experience of facilitating group exercises with their patients, and they noticed that the patients found value in group exercise. In workshop 3, the physiotherapists also suggested that initiating the exercise programme with a high frequency of instructor-led group exercise could potentially simplify the transition for patients to continue the exercise independently at home:

*“I’m also thinking that it would be really beneficial to have been in a group for a bit and know*,* having stood in the square*,* then it’s easier*,* and having trained a few times*,* to do it*,* transfer it*,* to when I’m at home exercising by myself. Something like that*,* pedagogically.”* (physiotherapists, workshop 3).

Furthermore, the physiotherapists reasoned that the combination of supervised exercise and the transition to unsupervised home-based exercise could be effective from an economical point of view and manageable in clinical work.

### To maintain motivation through meaningful exercise and structure

In terms of maintaining motivation for continued exercise, the older participants proposed several strategies, and they deliberated on whether motivation could be learned or developed in the absence of initial intrinsic motivation. In addition, the importance of discipline was highlighted:

*“There are many solutions*,* but it always requires self-discipline*,* that you do it and that you don’t trick yourself.”* (male older participant, workshop 2).

Discipline and motivation were identified as potential attributes that individuals could possibly acquire through strategic learning, although the older participants acknowledged this could be challenging. Life events, they noted, can impact one’s motivation to exercise. For instance, an older participant who had been exercising their entire life experienced a decline in motivation following a traumatic life event, taking several years to resume their exercise regimen. Therefore, the importance of individualised programmes and follow-ups was emphasised, as individuals require varying types of motivational support at different times.

The older participants’ preferences regarding exercise setting and prescription also influenced their motivation. While some preferred to exercise at home, others found that changing the setting enhanced their motivation. They also expressed the importance of appropriately challenging exercises. If the exercise is too demanding, some of the older participants experienced a loss of motivation. Furthermore, some of the older participants appeared to derive motivation from the performance of the exercise itself, but other older participants believed that a greater meaning or purpose behind exercising could serve as a motivating factor. For example, one male older participant noted that engaging in exercise could help them gain the confidence needed to resume other activities that had been previously discontinued:

*“…well-being depends on so many things*,* it’s not just about having balance*,* but also when you dare to get up and dance with her.”* (male older participant, workshop 4).

To sustain patients’ motivation, to continue exercising and allow progress, the physiotherapists also emphasised the importance of regular, individualised follow-ups. Furthermore, the physiotherapists expressed concern that without follow-ups, there was a risk of diminished patient motivation:

*“But someone who checks how it´s going at least*,* even if you’re exercising by yourself. Someone checks up on whether it’s working well for you now*,* and someone who checks. You know yourself when you´re going to exercise on your own*,* it easily falls through the cracks.”* (physiotherapists, workshop 5).

The physiotherapists highlighted the challenges of implementing consistent follow-ups in a clinical setting. They expressed frustration over obstacles such as limited resources, which hinder more intensive and sustained rehabilitation, and coordination issues among different healthcare providers. They emphasised that these follow-ups are crucial for patients’ motivation.

### To establish routines requires extra support

The older participants believed that incorporating exercise into daily routines was essential for consistency. However, they struggled to independently establish a routine due to the overwhelming number of available exercise programmes in general, the variety of exercises, and uncertainty about the optimal time for practice. Some older participants expressed difficulty in maintaining discipline to exercise alone at home, while this was preferred by others. One female described her morning routine getting ready for a morning exercise show on the television:

*“I also believe in scheduling. What´s motivating*,* and actually motivates me to get up in the morning*,* is that I want to make sure that I have time to have breakfast*,* get dressed*,* and be ready for 10 o’clock. Every morning*,* and I am.”* (female older participant, workshop 4).

Additionally, older participants suggested that support from family, friends, or health professionals could aid in establishing exercise routines. The physiotherapists deliberated on their professional role in supporting patients to establish a routine. They emphasised the importance of collaboration between different healthcare providers. A collaborative approach could streamline the implementation of a novel exercise programme and simplify the development of supportive strategies for patients.

## Discussion

This study explored the preferences and reflections of older people and physiotherapists during the co-creation of a motor–cognitive exercise programme to prevent falls. The findings highlighted several factors that warrant consideration. Initially, the concept was unfamiliar to the older participants, but through practical application it gradually became more relatable to their daily activities and led to new insights into their individual abilities, including their limitations. Based on older participants’ and physiotherapists’ experiences and preferences, we found key considerations for developing a motor–cognitive exercise programme include devising methods for effective exercises that are also safe; for example, by starting out in a group and graduating to home-based exercise. Additionally, individually tailored exercises and progression were described as essential, considering not only the variability and complexity of the population but also the motor–cognitive exercises. Furthermore, the older participants and physiotherapists highlighted meaningful strategies to foster motivation and compliance; for example, group exercise, establishing routines, and the provision and availability of continued support.

Both the older participants and the physiotherapists found motor–cognitive exercises to be enjoyable and challenging. The physiotherapists also perceived such exercises as somewhat complex to implement in practice. This perception may reflect the variability in exercise execution observed in previous studies in the field [15], underscoring the importance of future research into clinical application. Furthermore, the physiotherapists expressed concerns about balancing challenge and safety, describing their patients as fragile and emphasising the risk of potential injuries. In contrast, our study found that the older participants took responsibility for their own safety while exercising and expressed a willingness to learn how to perform exercises safely. This eagerness to understand safety and fall risk has been similarly reported in previous research [3]. Thus, while safety is a crucial consideration when developing challenging balance exercises, it may be important to not undermine older individuals´ ability to participate in risk analyses concerning their safety, providing they have had the right education and support to do so.

Tailoring to ensure appropriate exercise progression was identified as important by physiotherapists, and the complexity of performing and progressing motor–cognitive exercises was acknowledged, a challenge also noted in previous studies [32–34]. Our findings emphasise the heterogeneity of older people, which calls for a person-centred approach in exercise programme development and delivery [9]. For instance, the older participants in our study clearly preferred digital solutions over paper-based programmes, while physiotherapists observed varied preferences among their generally more frail patients, which may be related to this population’s health barriers [35]. Preferences and values regarding motivational factors and the meaningfulness of exercise also varied considerably among the older participants, and over the life-course, which is in line with previous research and theoretical models regarding meaningful activities [20]. Identifying the activities considered meaningful for individuals and perceiving an activity’s value have been described as important for both meaning and health [20, 36–38].

Many of the older participants in our study found group exercise to be both enjoyable and motivating. Within motor–cognitive exercise, there is a paucity of studies utilising group exercise and a limited evaluation of the group exercise setting [15]. However, previous research on physical exercise in general support these findings, indicating that group exercise can increase adherence among community-dwelling older people [39]. Additionally, our participants found instructor-led group exercise to be safe and meaningful, valuing it not only for the physical exercise but also for social interaction. Similarly, thematic synthesis of qualitative literature indicate that facilitators of physical exercise among older people included group exercise, which fostered a sense of belonging and enjoyment, while tailored, instructor-led exercise was valued and perceived as safe [40]. Furthermore, in our study, physiotherapists also recommended group exercise as a strategy for promoting safe exercise progression.

## Conclusion

This study explored perspectives of potential end-users during the co-creation of a motor–cognitive exercise programme for fall prevention. Initially unfamiliar, the motor–cognitive exercise concept became meaningful through practical engagement in activity, revealing individual abilities and limitations. Key considerations include ensuring safety, tailoring exercises to individual needs, and strategies to enhance motivation and compliance, such as group exercise, supervision, and support. These findings can inform the design of feasible and safe motor–cognitive exercise programmes, and represent a preliminary step towards a larger trial to evaluate their effects.

## Supplementary Information

Supplementary Material 1

Supplementary Material 2

## Data Availability

The datasets generated and analysed during the current study are not publicly available to protect the participants’ confidentiality (in accordance with The General Data Protection Regulation, European Union Regulation) but are available from the corresponding author upon reasonable request.

## Abbreviations

PAAR: Participatory and appreciative action and reflection
COREQ: The consolidated criteria for reporting qualitative research
IADL: Instrumental activities of daily living (IADL)
SD: Standard deviation
