# Supplementary material for: Co-creation of a motor–cognitive exercise programme—a qualitative study with older people and physiotherapists
Source: BMC Geriatr. 2025 Oct 15;25:780. doi: 10.1186/s12877-025-06522-3 (PMC12523024; doi:10.1186/s12877-025-06522-3)
Supplement: Supplementary file 2 — Supplementary Material 2 [file 12877_2025_6522_MOESM2_ESM.docx]

**Additional file 2**

| **Subcategory** | **Category** | **Theme** |
| --- | --- | --- |
| New to motor-cognitive exercise | To grasp a new concept can be challenging | Discovering the motor–cognitive concept through engagement in activity |
| Aware of motor-cognitive exercise but limited prescription experience |  |  |
| Difficult to describe motor-cognitive exercises |  |  |
| Able to identify and incorporate motor-cognitive exercises into daily life |  |  |
| Motor-cognitive exercise is considered as a challenge to perform | Practice leads to enlightenment |  |
| Motor-cognitive exercise is experienced as enjoyable and meaningful |  |  |
| Exercise with external stimuli distracts and feels difficult |  |  |
| Balance exercise should be challenging | To effectively challenge balance is inherently unsafe | Balancing safety and challenge |
| Challenging exercise feels positive |  |  |
| Variation in individual perception of safety | Safety is a crucial aspect |  |
| Instructor led exercises is needed for safety |  |  |
| Safe home exercise comes at the cost of effective balance exercise |  |  |
| To safely challenge balance at home is difficult in practice and requires personal responsibility. |  |  |
| Awareness that all kinds of exercise does not apply to all older people | It is important to recognise variability within the older population | Navigating the complexity of individualisation |
| What feels fun can change during a lifespan |  |  |
| The exercise programme and follow- up should be individual adapted |  |  |
| Digital tools with clear and accessible instructions preferred over paper formats |  |  |
| Need for guidance to ensure correct performance | Multiple components complicate progression |  |
| Progression of motor-cognitive exercises is important yet challenging |  |  |
| Follow-up could be managed in a group setting |  |  |
| Group exercise gives social motivation, support and feels enjoyable | To exercise together for enjoyment and social engagement | Managing motivation and compliance |
| Leader led group exercise provides emotional and psychological comfort |  |  |
| Group exercise offers structural, practical and economic benefits |  |  |
| Preferences of exercise settings vary and influences motivation to exercise | To maintain motivation through meaningful exercise and structure |  |
| Motivation decreases when tasks are overly challenging |  |  |
| Greater meaning of well-being behind exercise performance- a motivational factor |  |  |
| Life- turning events affect motivation and discipline to exercise |  |  |
| Loved ones can support exercise participation | To establish routines requires extra support |  |
| Exercise needs to become a regular routine |  |  |
| Exercising alone at home and a wide selection of exercises, hinder establishing routines. |  |  |
| Collegial collaboration strengthens implementation |  |  |

**Table 1.** More detailed description of the analysis procedure.
